# Ten simple rules for scientific code review

**DOI:** 10.1371/journal.pcbi.1012375

**Published:** 2024-09-05

**Authors:** Ariel Rokem

**Affiliations:** Department of Psychology and eScience Institute, University of Washington, Seattle, Washington, United States of America; Dassault Systemes BIOVIA, UNITED STATES OF AMERICA

## Abstract

As large, high-dimensional data have become more common, software development is playing an increasingly important role in research across many different fields. This creates a need to adopt software engineering practices in research settings. Code review is the engineering practice of giving and receiving detailed feedback on a computer program. Consistent and continuous examination of the evolution of computer programs by others has been shown to be beneficial, especially when reviewers are familiar with the technical aspects of the software and also when they possess relevant domain expertise. The rules described in the present article provide information about the why, when, and how of code review. They provide the motivation for continual code reviews as a natural part of a rigorous research program. They provide practical guidelines for conducting review of code both in person, as a “lab meeting for code,” as well as asynchronously, using industry-standard online tools. A set of guidelines is provided for the nitty-gritty details of code review, as well as for the etiquette of such a review. Both the technical and the social aspects of code review are covered to provide the reader with a comprehensive approach that facilitates an effective, enjoyable, and educational approach to code review.

## Author summary

Scientists are increasingly writing code as part of their research. Code review is a common practice in software engineering, which entails detailed and continual examination of additions and changes to a software code-base. This article explains why and how this practice is applied to the software that researchers write as part of their work. It provides a set of rules that motivates, explicates, and details the process of using code review in a didactic, effective, and enjoyable manner.

## Introduction

The availability of large, heterogeneous, and noisy datasets has led to the adoption of data-intensive research methodologies in many fields that previously did not experience such datasets. For example, computer programming, which was once a tool mostly used by a small group of experts, is now part of many scientists’ toolbox [1]. The spread of programming has increased the need to adopt software engineering practices into many different research settings [2,3].

The present article focuses on code review, a software engineering practice that has the potential to expose software errors early in the research process, increasing the veracity and reproducibility of research results that depend on software. It can also benefit the training and professional development of researchers who are still honing their programming craft. Code review is a very common practice among professional software developers and software engineers. In these professional settings, it has been directly linked to code quality [4,5]. For example, McIntosh and colleagues [5] showed that increased review coverage (i.e., how much of the code gets comments in the review process) can increase code quality (in terms of errors that make it into released code and need to be subsequently fixed). In addition, how much discussion a code review entails also increases code quality. Code reviewer expertise (e.g., understanding of the research domain) is also a factor that contributes to code quality. When practiced in scientific research settings, it has also been demonstrated to yield benefits in terms of accuracy, efficiency, and the adoption of better software practices [6]. Fortunately, based on its common use in software engineering, there is a large body of research literature that addresses the questions about the most effective approaches to code review. The brief set of rules presented below aims to introduce scientists from a broad range of backgrounds to code review, suggesting a few best practices taken from this research and incorporating some of the author’s experience conducting code review in various scientific settings.

## Rules for code review

### Rule 1: Review code just like you review other elements of your research

The first step in adopting code review practices is to recognize the importance of this practice. If you are working with collaborators, students, or supervisors who have not conducted code review before, you may need to advocate for it. This paper provides references to some of the empirical evidence in favor of code review. Another important point of view is that reviewing code is equivalent to the many other review procedures that accompany a research project. For example, no reasonable scientist would submit a paper manuscript that has not been thoroughly checked by their coauthors. In the process of writing, we expect our collaborators to examine the flow of a paper and its logic, as well as to check the clarity, grammar, and syntax of the written text. Code, while not always a public-facing element of the research, should be submitted to (at least) a similar level of scrutiny. Once submitted to such scrutiny by mentors and collaborators, its publication together with the paper could become both psychologically and technically easier. This has the added benefit that the research becomes more reproducible [7].

### Rule 2: Don’t leave code review to the end of the project

In data-intensive research projects where software is involved, the correctness of the result depends on the correctness of the software (prominent counterexamples are fuel for computational scientists’ nightmares [8]). This suggests that a substantial cost can be mitigated by building continuous review rounds into the research process from its very beginning. Furthermore, because code review is most effective when done in limited chunks (see point 8), it benefits from committing a little bit of time on a regular basis [6]. In addition, just like experimental research benefits from early feedback, to work out the issues with experimental design and assure that alternative explanations are properly controlled, analysis software can benefit from early feedback, even before it is entirely complete. Reviewing code continuously throughout the life of a research project also helps avoid the accrual of “technical debt” [9]. These are the aspects of the code that researchers refer to when you ask them to show you their code and they say something like “I just need to clean it up a bit.” A corollary of rules 1 and 2 is that code review is an important element of training junior programmers and to the extent that all data-intensive research now involves programming, it has become an important part of the training of junior scientists, just like journal clubs and scholarly peer review.

### Rule 3: The ideal reviewer may be closer than you think

The scientific context and premise of your research are crucial to understanding what your code does. One of the findings of [5] was that “reviewer expertise has a measurable impact on post-release defect counts in the larger studied releases.” This means that rather than supporting the notion that some external expert in software engineering would be the best reviewer of your code, these results suggest that appropriate reviewers—that can help reduce errors in your code—are close by, in your lab mates, collaborators, mentors, and students. In fact, the best reviewer of your code may be even closer than that, as it is often a good idea to read carefully through your own code and think it through properly before submitting it for review by others. As in the case of written notes, the fact that someone else will read your code is likely to make your reading of the code more critical and will already likely lead to improvements.

### Rule 4: Make it easy to review your code

One of the main barriers to effective code identified in Petre and Wilson’s study [6] was that the code was not properly documented and lacked automated build tools and example datasets. Making your software easy to install and run, providing example data, and rudimentary documentation will make it easier to understand and to provide useful feedback. These steps also increase the value of the software, as they can potentially make the software reusable by others. In fact, code that is used and reused should not remain in scripts or computational notebooks but rather be organized in reusable and testable libraries, where it is also easier to review the individual functions and modules. Instructions on how to create a software library are beyond the scope of the present paper and the interested reader is referred to resources for the commonly used Python ([10]; Chapter 7) and R [11] programming languages. With regard to sharing of example data: share as much as is practical and useful. Comprehensive and complete example datasets provide a sense of what is possible and expected with a particular software package and provide the basis for reproducing or building upon your results. On the other hand, reducing friction in understanding the software is a high priority as well, and it should not entail downloading and storing very large amounts of data, or waiting for long-running processing pipelines. For example, in neuroimaging software that my group and I develop [12,13], we provide functions that download test datasets and datasets that serve as the basis for documentation examples. In some cases, we provide data that have only been minimally preprocessed, demonstrating end-to-end functionality of the software, but because neuroimaging data can be rather large and processing can be time-consuming, we also sometimes provide fetching functions for data that have been partially processed (along the lines of a cooking show). These partially processed data allow us to demonstrate or test particular functionality that depends on data that have already undergone multiple time-consuming steps.

### Rule 5: Do it in person and synchronously…

One way to conduct code review is by looking at code together side-by-side. In many contexts, a “lab meeting for code” is a good idea (I first learned about this idea from Fernando Pérez, who also wrote a blog post that lays out this idea and describes the provenance of this term: https://web.archive.org/web/20170701202441/http://fperez.org/py4science/code_reviews.html). To implement this idea, you would:

- **Circulate code in advance**: Code can be sent to all participants or posted to an online repository (e.g., in GitHub). To facilitate a productive synchronous code review session, code would be circulated well in advance, allowing attendees time to examine the code in advance and prepare their comments.
- **Provide necessary context**: For a review to be fruitful, some of the scientific context is required. When conducted within a close-knit group, such as a lab, it is possible that many of the attendees are already familiar with the scientific context, in which case maybe only a quick introduction to the specific context of the software (e.g., 1 or 2 slides) may be needed. One important piece of context is whether the code that you have written is intended as a tool for use by others, or whether it serves only for the specific requirements of a project (a “one-off”). Both are legitimate objects for code review, but the purpose of the review and the points for emphasis (e.g., the thoroughness of documentation) may vary.
- **Ask for specific feedback if needed**: Explain what you expect to get from the review. Point out what kind of feedback would be most useful for you. Maybe you are struggling with the performance of a particular section of your code, or maybe you want to make sure that you are correctly using some tool. Maybe you want another set of eyes on the implementation of a particularly complicated mathematical equation. Since your time in such a meeting is necessarily limited, provide your priorities so that the most pressing needs are addressed first.
- **Walk through the code**: As in other cases in which lab meeting presentations are done, the code author projects their code onto a screen (or shares their screen with others through a teleconference system) and presents the code. The presentation can proceed line by line or, better yet, by taking the attendees through the logical order of code execution. Walking others through the logic of the code can help them understand what the code is supposed to do. The detailed walk-through is one of the main benefits of the synchronous in-person format, as it allows attendees to ask clarification questions and provides an opportunity for dialogue.
- **Gather actionable comments and suggestions**: One effective way to gather the comments and suggestions provided by attendees is to annotate the code with in-line comments. A subsequent revision of the code can then address the comments wherever they are most relevant.

### Rule 6:…and also remotely and asynchronously

Asynchronous, remotely conducted code review is the standard operating procedure in many open-source scientific software projects. Many tools support asynchronous code review, but the tool that is most commonly used in these projects is the GitHub “pull request” (PR) interface [14]. This interface allows a programmer to introduce a set of changes into existing software (requesting that someone else “pull” these changes into the main branch of the software development repository). Other contributors to the software can then provide line-by-line comments on the code and propose specific changes that can be immediately integrated into the PR. Additional tools within the GitHub interface provide the ability to automatically check the code for its compliance with project coding standards (e.g., the PEP8 coding standard for the Python programming language, as implemented in the flake8 software, which also checks the code for “flakiness,” such as unused imports; https://flake8.pycqa.org/en/latest/), and even to automatically run a software test suite [15]. One way for students to receive code review from domain experts who also have software engineering expertise and to improve their programming is to propose and contribute changes to an open-source software project within their field. Many scientific software projects participate in software internship programs, such as Google’s Summer of Code internship (https://summerofcode.withgoogle.com/), providing students an opportunity to gain experience and also receive continuous and consistent review of their code contributions. One of the benefits of asynchronous code review is that recording actionable comments and suggestions is built into the process. Furthermore, the process provides a persistent record of the review, and the comments and suggestions provided through this process can be referred to in the future, to understand why certain changes were made.

### Rule 7: Review systematically

There are many ways to review code, and code review will vary slightly depending on the stage of a project, and depending on the type of feedback needed. One systematic approach is to review the code in several iterations, focusing on different aspects of the code in different passes through, starting from high-level design and execution, and then focusing on specific low-level aspects.

- **Run the code and aim to reproduce the results**: The first part of a review of research software can also serve as a “reproducibility audit” for the research results [16]. The person reviewing the code tries to follow the instructions provided to install and run the code and can verify that the results are independently reproduced (with some data; see rule 4). In asynchronous review settings, automated systems collectively known as “continuous integration” or “continuous deployment” can provide affirmative proof that the code installs and runs as expected.
- **Read the code through—first all of it, with a focus on the API**: If you are reviewing a large amount of code, get a sense of the intended purpose of the code first, before you dive into the details. Just like in a paper review, it is often useful to restate at the beginning of the review what you think the author intended to do in this work in a few sentences. This sets the stage for the review of the details. Reading the tests or documentation can give you a good sense of the intended use-cases and the API (application programming interface). This is often a good place to start, because comments about the API are often the ones that will require the most dramatic changes in the code, and you can save time for everyone involved by starting with those, rather than starting with requests to fix typos, which eventually get scuttled when the API changes dramatically.
- **Ask questions**: Asking questions is almost always a good idea. You want to make sure that you understand the intentions of the code author. This is particularly important when conducting code review in interdisciplinary research contexts, where researchers from different fields may have a different understanding of the meaning of certain concepts (for example, try asking a physicist, a mathematician, a psychologist, and a statistician what a “linear model” is).
- **Read the details—focus on modularity and design**: One of the main contributions you can make as a reviewer is to pull the code author “away” from the details, to point out the big picture of the code. It is quite usual, particularly for scientific code, that the software is implemented for a specific project or use case and fails to take into account a more general class of use cases. A different perspective can be very helpful. This might entail changes in the architecture of the code (this is sometimes known as “refactoring”), or in its broader design, to accommodate and enable use cases that were not on the code author’s mind when they initially wrote the software.
- **Read the details—focus on the math**: Scientific software often implements mathematical ideas. If it is called for, make sure that you understand the mathematical ideas that are implemented and the ways in which the math got transferred to code. From my own experience, this is one of the hardest things to do in review and a common source of error in writing code. Remember that tests and documentation are also code and need to be reviewed with utmost care for these kinds of errors, as they often serve as a benchmark or source of truth about the functionality of the software.
- **Read the details—focus on performance**: When going through the code on this pass, take a look at parts of the code that look like they might be performance bottlenecks. This is particularly easy if the code author has pointed out performance issues and potential bottlenecks. Consider what could be done to relieve these bottlenecks: Has the code author taken advantage of all the tools at their disposal? For example, many software languages used in scientific computing provide substantial advantages for the vectorization of mathematical operations that are repeated over homogeneously typed arrays. Languages may have specialized tools for speeding up slow performance-critical code, such as Cython [17] or Numba [18]. Note that adopting new tools can sometimes be cumbersome. Consider the costs and benefits of adopting a new tool when offering advice about how to improve performance.
- **Read the details—focus on formatting, typos, comments, documentation, and overall code clarity**: Finally, after all the other concerns have been alleviated, you can dive into the nitty-gritty: comment about choice of variable and function names, typos in the documentation and comments, and the format and clarity of the documentation (some of these issues will surface in the first stage, as they pertain to the overall clarity of the author’s intentions). Though you want to make sure that the code is properly formatted, that conventions of the language and the project are followed, and there aren’t any language errors, be sure to mark comments that are minor as such. It allows the author to address them, without stressing about them.

### Rule 8: Know your limits

Empirical studies demonstrate that review quality decreases precipitously when reviewing more than approximately 200 lines of code in one sitting [19]. The same research suggests that you should expect to spend about 60 minutes reviewing 500 lines of code and that you should take your time doing it. This means that you should take a break after about 30 to 60 minutes of reviewing code. This also sets a clear limit on how much code you can request others to review for you. These types of limitations can also affect the conceptual units into which software is divided, encouraging better modularity and separation of concerns, both of which make code easier to test, debug, and more general to use.

### Rule 9: Be kind

Many research fields, and the researchers within them, are still adjusting to the need to incorporate software development into their research practice. Software development is also an area that is particularly prone to imposter syndrome and stereotype threat [20–22]. Remember that the code was written by a person, who expended creative effort into writing the code, and may already be feeling vulnerable sharing the code with others and submitting their work to scrutiny. Do not be dismissive, even if this person did things that you consider to be grave mistakes. This can be particularly hard to remember when conducting asynchronous code review, without the other person’s immediate presence. A review that is delivered with kindness is more likely to be accepted with attention.

### Rule 10: Reciprocate

Much like one of the best ways to learn about your research field is to teach it to others, one of the best ways to become a better programmer is to read a lot of software written by others. Therefore, don’t hesitate to reciprocate and review other researcher’s code. If nothing else, you will be helping them root out errors and improve their software. In the best case, you might also learn some new tricks along the way. Needless to say, both being kind and reciprocating a review will also pay off in good karma.

## Conclusions

Adopting code review as a practice can yield benefits in many different research settings. Introducing this practice may sometimes meet with resistance. Especially in contexts in which it is not currently common. Nevertheless, researchers can motivate the investment of time and effort based on the extensive research that demonstrates the efficacy of the practice [4,5] and based on the potential cost of software errors in research [8]. The rules provided here provide both inspiration, as well as the nuts and bolts of code review in a research setting. The goal is that as code review becomes increasingly practiced in research settings, the quality of code and, as a direct consequence, the quality of research overall, will increase.
